# Development of an electrospun poly(ε-caprolactone)/collagen-based human amniotic membrane powder scaffold for culturing retinal pigment epithelial cells

**DOI:** 10.1038/s41598-022-09957-5

**Published:** 2022-04-19

**Authors:** Elahe Majidnia, Mehdi Ahmadian, Hossein Salehi, Noushin Amirpour

**Affiliations:** 1 Department of Materials Engineering, Isfahan University of Technology, 84156-83111 Isfahan, Iran; 2 Department of Anatomical Sciences, School of Medicine, Isfahan University of Medical Sciences, 81746-73461 Isfahan, Iran

**Keywords:** Medical research, Engineering, Materials science

## Abstract

The common retinal diseases are age-related macular degeneration (AMD) and retinitis pigmentosa (RP). They are usually associated with the dysfunction of retinal pigment epithelial (RPE) cells and degeneration of underlying Bruch’s membrane. The RPE cell transplantation is the most promising therapeutic option to restore lost vision. This study aimed to construct an ultrathin porous fibrous film with properties similar to that of native Bruch’s membrane as carriers for the RPE cells. Human amniotic membrane powder (HAMP)/Polycaprolactone (PCL) scaffolds containing different concentrations of HAMP were fabricated by electrospinning technique. The results showed that with increasing the concentration of HAMP, the diameter of fibers increased. Moreover, hydrophilicity and degradation rate were improved from 119° to 92° and 14 to 56% after 28 days immersion in phosphate-buffered saline (PBS) solution, respectively. All scaffolds had a porosity above 85%. Proper cell adhesion was obtained one day after culture and no toxicity was observed. However, after seven days, the rate of growth and proliferation of ARPE-19 cells, a culture model of RPE, on the PCL-30HAMP scaffold (HAMP concentration in PCL 7.2% by weight) was higher compared to other scaffolds. These results indicated that PCL-30HAMP fibrous scaffold has a great potential to be used in retinal tissue engineering applications.

## Introduction

Age-related macular degeneration (AMD) is the leading cause of visual impairment and blindness in individuals over 50^1^. Due to the increase of the aging population, the incidence and burden of AMD are expected to increase alarmingly in the coming years^2^. Retinitis pigmentosa (RP) prevalence is approximately 1 in 5000 individuals^3,4^. The diseases are characterized by the degeneration of a specific cell layer at the back of the eye, the retinal pigment epithelium, which is essential in retinal function^5^. So far, no efficient treatment has been provided for these diseases. Gene therapy and anti-angiogenic drugs just delay the progression of these diseases. One of the best treatments is to replace the destructed cells with new RPE ones^6,7^. Cell transplantation may have the potential for retinal regeneration. However, several problems hinder the successful repair of the retina including disorganized or misplaced grafts, as well as reflux of the cells from the injection site, which might lead to serious complications including RPE cell stacking, cell death, and retinal fibrosis. Recent studies have shown that the use of scaffolds can address these obstacles^8,9^. The RPE cells are located between the photoreceptors and the underlying Bruch’s membrane (BM) which separates the RPE from the blood vessels of the choroid. It is an extracellular matrix with a thickness of 2–4.7 µm that acts as a molecular sieve to maintain the metabolic exchange between the vasculature and outer retina^5,10^. Since in AMD the underlying BM is often compromised, the thin scaffold can further act as a prosthetic BM, ensuring the survival, integrity, and functionality of the attached RPE cell monolayer^5^. Various membranes have been used as scaffolds for RPE cells, but long-term cell viability and functionality are still largely unknown^9^.

As a biodegradable aliphatic polyester with high tensile and elongation properties, PCL has been widely used for tissue engineering applications because it was approved by the U.S. Food and Drug Administration (FDA) for specific applications in the human body. It produces neutral pH and non-inflammatory by-products. Furthermore, it is easy to process and can be fabricated in various shapes such as fibers, scaffolds, and membranes. Previous studies have shown that electrospun PCL scaffolds not only can support the growth and proliferation of retinal cells but also presented biocompatibility after subretinal transplantation in an animal model. However, slow degradation rate, inherent hydrophobicity, and inadequate cell affinity due to the lack of recognition sites for cell adhesion make it less ideal for use as a prosthetic BM material^8,9^. The human amniotic membrane (HAM) has many advantages that make it an applicable biomaterial. The extracellular matrix of amnion is composed of different collagen types (I, III, IV, V, and VII), laminin, and fibronectin similar to BM, and its availability is virtually unrestricted. As a biological tissue material, HAM has low immunogenicity and to date, it has been widely adopted in clinical practices. However, some features such as low biomechanical consistency and rapid biodegradation have limited its application^11^. Consequently, blending the bioactive functions of HAM with the good mechanical properties of PCL for the generation of new bio-hybrid material could show an improvement in biological, mechanical, and degradation properties compared to the individual components. The critical influence of fiber size on cellular performance has led to the use of different solvent systems for the production of specific nano- or micro-sized PCL fibers while neglecting the toxicity of the solvent. The goal is to use the unconventional solvent system acetic acid/formic acid with very low toxicity, recently defined as the system producing ultra-thin PCL fibers^12^.

In this study, an ultrathin and porous fibrous membrane composed of PCL and HAMP was fabricated by electrospinning to replicate the BM. To evaluate the effects of HAMP on the scaffolds, the structural, physical, and mechanical properties as well as in vitro degradation behavior of scaffolds and human RPE (hRPE) cells behavior on them were investigated.

## Materials and methods

### Materials

PCL (Mw, 80 kDa) was purchased from Sigma–Aldrich Co. (St. Louis, MO, USA). Formic acid (FA) and acetic acid (AA) were also bought from Merck Co. (Darmstadt, Germany). Human placentas were taken from the cesarean section of Milad hospital (Isfahan, Iran). ARPE-19 cells were purchased from the Isfahan Royan Institute (Isfahan, Iran). Dulbecco’s modified Eagle medium (DMEM (+) Glutamax. High glucose), fetal bovine serum (FBS), trypsin/EDTA, phosphate-buffered saline (PBS), penicillin–streptomycin, and MTT (3-(4,5-Dimethyl-2-thiazolyl)-2,5-diphenyl-2H-tetrazolium bromide) were obtained from Bio-Idea (Tehran, Iran).

### The procedure of HAMP preparation

**Figure 1:**
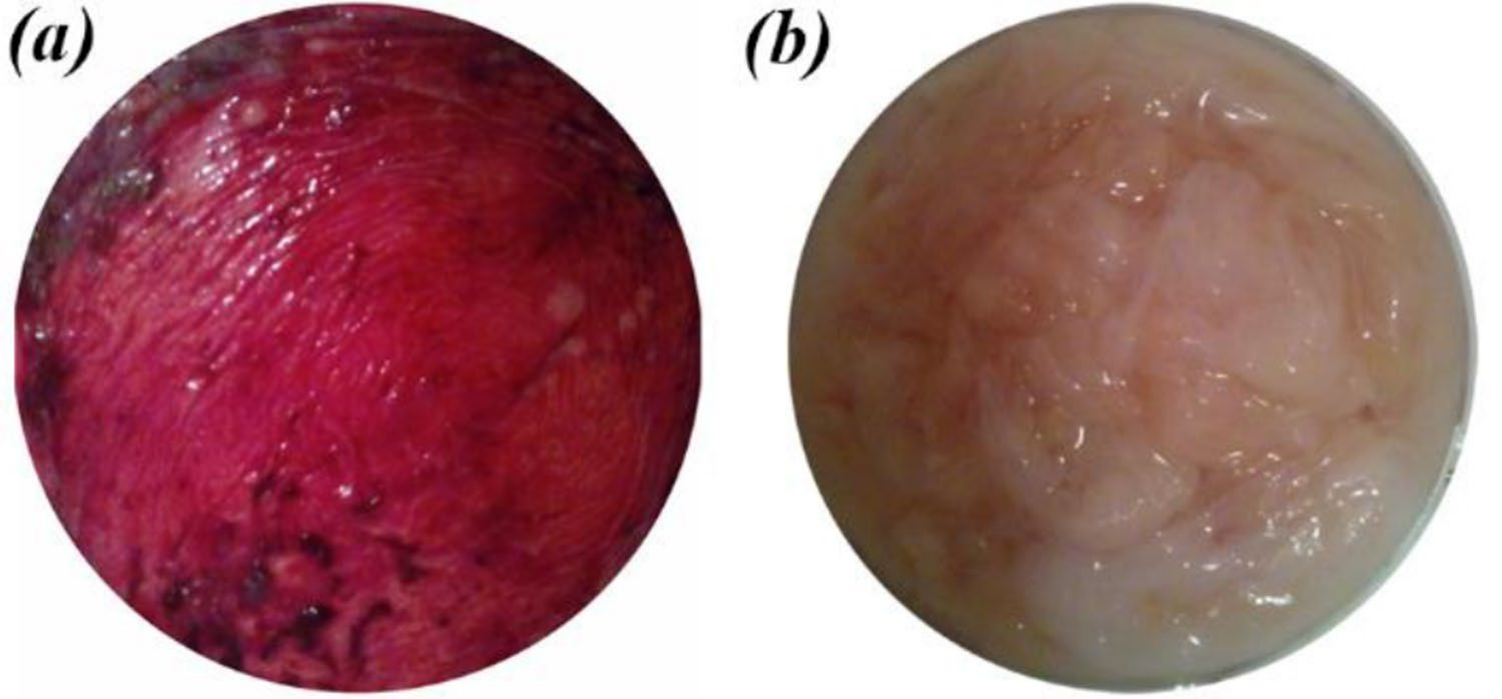
Human amnion membrane (**a**) before washing, (**b**) after washing.

For the preparation of HAMP, the human placental tissues were obtained from healthy donor mothers (n = 7; 35–45 weeks’ gestation), who provided informed consent. All experiments and protocols in this study were performed in accordance with the declaration and approval of the Isfahan University of Medical Science (IUMS) Ethics committee and according to IUMS policies on medical and research ethics (approval ID: IR.MUI.RESEARCH.REC.1398.148). The amnion membranes were manually dissected from the chorion membranes. Any blood clots were removed using a sterile forceps. The membranes were washed with sterile saline. Using sterile scissors and forceps, the amnion membranes were cut into approximately 5 × 5 cm pieces. The amnion pieces were washed with sterile saline and then with sterile water. The amnion pieces were transferred into a Petri dish (Fig. 1). The Petri dishes containing the amnion pieces were then kept at − 80 °C for 24 h. The Petri dishes were placed in a precooled glass lyophilizer container and were lyophilized for 48 h. Then, the lyophilized amnion membrane pieces were ground into powder using a ball mill with zirconia vial and balls (Retsch, PM100, Germany) at ambient temperature.

### Characterization of HAMP and the solvent effect

To characterize the amniotic membrane powder and the effect of the solvent on it, Fourier transform infrared spectroscopy (FTIR, Bruker, tensor27, Germany) of the amniotic membrane powder and the resulted film from its dissolution in the solvent system FA/AA (70/30) were performed over a range of 300–8000 cm^−1^ and the resolution of 2 cm^−1^. To prepare the amniotic membrane film, HAMP solution with a concentration of 5% wt./v in the above solvent system was prepared and after 24 h of mixing with a magnetic stirrer, it was poured into a Petri dish and the obtained solvent was removed under the hood for 48 h. The resulting film was then sent for infrared spectroscopy.

### Measurement of the solution properties

The viscosity of the solutions was measured at 25 °C using a viscometer (Brookfield, DV-II + pro, USA). The changes in electrical conductivity due to the various concentrations of the polymeric solutions and the weight ratio of HAMP to PCL were determined at 25 °C using a conductivity meter (Inolab, Cond 7110, Switzerland).

### Electrospinning of the PCL and PCL/HAMP scaffolds

Before electrospinning, PCL/HAMP solutions consisting of various amounts of HAMP were prepared. At first, 5% (wt./v) HAMP stock solution in FA/AA with a volume ratio of 70/30 was prepared and stirred for 24 h. It is worth mentioning that this concentration was selected based on its ability to cross the stainless steel 23-gauge needle and this solvent mixture was selected based on its ability to provide finer fibers^13^. On the other hand, 16, 17.6, 19.2, 20.8, 22.4, and 24% (wt./v) PCL solutions in a mixture of FA/AA with a volume ratio of 70/30 were prepared separately. To obtain various concentrations of HAMP in PCL (0, 2.8, 5.2, 7.2, 8.9 and 10.4 wt. %.), stock HAMP solution was added to the PCL solutions in the ratios of 0:100, 10:90, 20:80, 30:70, 40:60, and 50:50 (v/v), respectively, and then stirred to prepare homogenous solutions. The concentration of the PCL solutions was selected in a way that after adding the HAMP solution, PCL concentration in the final solution is 16% (wt/v). The samples with different volume persent of HAMP were labeled as PCL, PCL-10HAMP, PCL-20HAMP, PCL-30HAMP, PCL-40HAMP, and PCL-50HAMP, respectively.

The electrospinning apparatus employed in this study contains a syringe pump (Syringe Pump, DAIWHA, MS-2200, Korea), a high voltage supply (Voltage Regulator, Gold Star, Korea), and a collector covered with aluminum foil. The electrospinning process was performed with a set of optimized parameters. A voltage of 20 kV was applied to a syringe tip, while the collector was grounded. The polymer solution was loaded into a 1 mL disposable syringe with a blunt-end needle (23-gauge) which was controlled by a syringe pump at a feeding rate of 0.5 mL/h. The distance between the needle tip and the collector was adjusted to 12.5 cm. A charged jet of the solution was formed and ejected towards the collector, during which time the solvent evaporated and the fibers were deposited on the surface of the collector to form a flat fibrous membrane. The thickness of the electrospun membrane was controlled through the length of the processing time. All the electrospinning experiments were performed at room temperature and humidity. The as-spun fibers were dried in a vacuum desiccator for at least 24 h at room temperature to dissipate the remaining solvent before characterization.

### Structural, physical and mechanical characterizations of the scaffolds

The morphology of fibrous membranes was examined using scanning electron microscopy (SEM, Philips, XL30, Netherlands). Before SEM imaging, the samples were coated with a thin layer of gold. The diameter of the electrospun fibers was determined by measuring about 100–120 individual fibers with the Digimizer software, and the histogram charts of their distribution were drawn using the Minitab software. Furthermore, Fourier-transform infrared spectroscopy (FTIR, Bruker tensor) was performed over a range of 500–4000 cm^−1^ and a resolution of 2 cm^−1^.

To measure the porosity, the membranes were put into a pycnometer full of alcohol. The membranes were taken out, and the pycnometer and the remaining alcohol were weighed. The porosity of the membranes was calculated using the following equation:^9,14^

$$Percentage\;of\;prosity=\frac{{W}_{2}-{W}_{3}-{W}_{s}}{{W}_{1}-{W}_{3}}\times 100$$

where W_1_ is the weight of the pycnometer that is filled with alcohol, W_2_ refers to the weight of pycnometer with alcohol and scaffolds, W_3_ is the weight of pycnometer and alcohol after removal of the scaffolds, and W_S_ is the initial weight of the membranes in a dry state.

The wettability of the electrospun membranes was evaluated through water contact angle analysis by a sessile drop method using 4 mL deionized water that was dropped onto the surfaces of fibrous membranes. After 10 s, the shapes of the water droplets were recorded by an optical contact angle meter system (Sharifsolar, CA-500, Iran). Each determination was repeated three times in different positions. The values of the contact angle were expressed as the mean ± SD.

Mechanical properties of the membranes were explored by applying a tensile test to all of the specimens prepared from the scaffolds using a materials testing machine (Hounsfield, H25K-S, England) at an elongation speed of 2 mm/min. Young's modulus was calculated by measuring the slope of the initial linear region of the stress–strain curves. The thickness of the fibrous scaffolds was measured by a micrometer (HD Digital Outside Micrometer 0-25 mm/0.001 mm).

### Biodegradability

To observe the biodegradability of prepared scaffolds, each sample (n = 3) of the known mass (w_0_) was immersed in PBS and maintained for 3, 7, 14, and 28 days at 37 °C. In the specified days, the scaffolds were removed, washed 3 times with distilled water, dried, and weighed (w_t_). The percentages of weight loss were calculated from the equation described below^15,16^:

$${\mathrm{W}}_{\mathrm{tloss}}\mathrm{\%}=\frac{{\mathrm{W}}_{0}-{\mathrm{W}}_{\mathrm{t}}}{{\mathrm{W}}_{0}}\times 100$$

where W_tloss_% is the weight loss percentage of the sample after time t, W_0_ is the sample weight at the beginning of the degradation test, and W_t_ is the sample weight after time t.2.8. Cell culture, viability, and morphology study.

The human retinal pigment epithelial cell line (ARPE-19) was used in these experiments. The cells were characterized by using immunocytofluorescence staining and RT-PCR for gene expression. Immunocytofluorescence staining was carried out as the standard protocol using the antibodies ZO-1(Invitrogen 339100) and Cytokeratin 18 (Chemicon, MAB3234). Labeled cells were visualized with TRITC-conjugated secondary antibodies (TRITC anti-mouse IgG, Sigma-Aldrich, T7782), and nuclei were stained with 4′,6-Diamidino-2-phenylindole dihydrochloride (DAPI) (Sigma, D8417). Stained cells were observed under a fluorescence microscope (Olympus, BX51, Japan). For RT-PCR analysis, the total RNA was isolated by a high pure RNA Isolation Kit (Roche) according to the manufacturer's instructions. The RNA was reverse transcribed using RevertAid First Strand cDNA Synthesis Kit (Thermo Scientific) with oligo dT primers. RT-PCR was performed by 2 µg total RNA and GAPDH was used as a loading control. The primer sequences and accession numbers are shown in Table 1.

**Table 1:** PCR primer sequences used for RT–PCR.

mRNA	Forward primer (5′-3′)	Reverse primer (5′-3′)	Accession number	Size	Tm
CRALBP	CCTCAACTGTCCTGGACCCAAGGC	TTACCCATCCCCCAACTTGAGA	NM_000326	123	58
RPE-65	GCCCAGGAGCAGGACAAAAG	GCGCATCTGCAAGTTAAAACCA	NM_000329	247	52
GAPDH	CCCCACCACACTGAATCTCC	GGTACTTTATTGATGGTACATGACAAG	NM_002046	104	60

The cells were cultured in DMEM (+) Glutamax, high glucose medium supplemented with 10% fetal bovine serum (FBS), 1% non-essential amino acid (NEAA), and 1% penicillin/streptomycin^17^. The cells were grown in 75 cm^2^ tissue culture flasks and maintained in an atmosphere containing 5% CO_2_ and 80% humidity at 37 °C. The medium was replaced every three days throughout the experiments. All the scaffolds were sterilized by immersion in 70% ethanol for half an hour and then were exposed to UV radiation for both top and bottom surfaces in a laminar flow hood (each surface for 30 min). After reaching about 80% confluence, the cells were detached by 0.05% trypsin/EDTA. A density of 10,000 cells/cm^2^ was seeded onto the scaffolds in 24-well plates. In all the experiments, ARPE-19 cells from passages 5 to 20 were used.

To investigate the viability of ARPE-19 cells on the scaffolds, an MTT assay was performed after 1, 3, and 7 days of cell culture in 24-well plates. Tissue culture polystyrene (TCP) was served as control. After each time point, the samples were washed with PBS and incubated with MTT (5 mg/ml) reagent in a serum-free culture medium at 37 °C in an atmosphere containing 5% CO_2_ and 80% humidity. The MTT was reduced to formazan by mitochondrial enzyme dehydrogenase in viable cells. After 4 h of incubation, the formazan crystals were dissolved in DMSO for 10 min and then aliquots were pipetted into 96-well plates. The absorbance of the content of each well, formazan in the cell culture, was measured at 545 nm using a Microplate reader (Hiperion, MPR4 + , Germany). The amount of formazan is directly proportional to the number of viable cells.

The morphology of the grown cells on fibrous scaffolds was observed by SEM (Philips, XL30, Netherlands). After 1 and 7 days of cell seeding, the cells were fixed by glutaraldehyde solution (3%, Merck) in PBS for 30 min at room temperature. The dehydration process was performed on each specimen in a series of graded alcohols (50%, 70%, 80%, 90%, and 100%), each for 10 min. They were then sputter-coated with gold and viewed by SEM.

### Statistical analysis

All the data presented in this paper are shown as the mean ± standard deviation (SD) unless otherwise indicated. All the experiments were repeated at least three times. The data from the experimental groups were compared with those from the controls. Statistical analyses of the obtained data were performed by one-way ANOVA, and the values of P ≤ 0.05 were considered statistically significant.

## Results and discussion

### FTIR studies of HAMP before and after the dissolution

**Figure 2:**
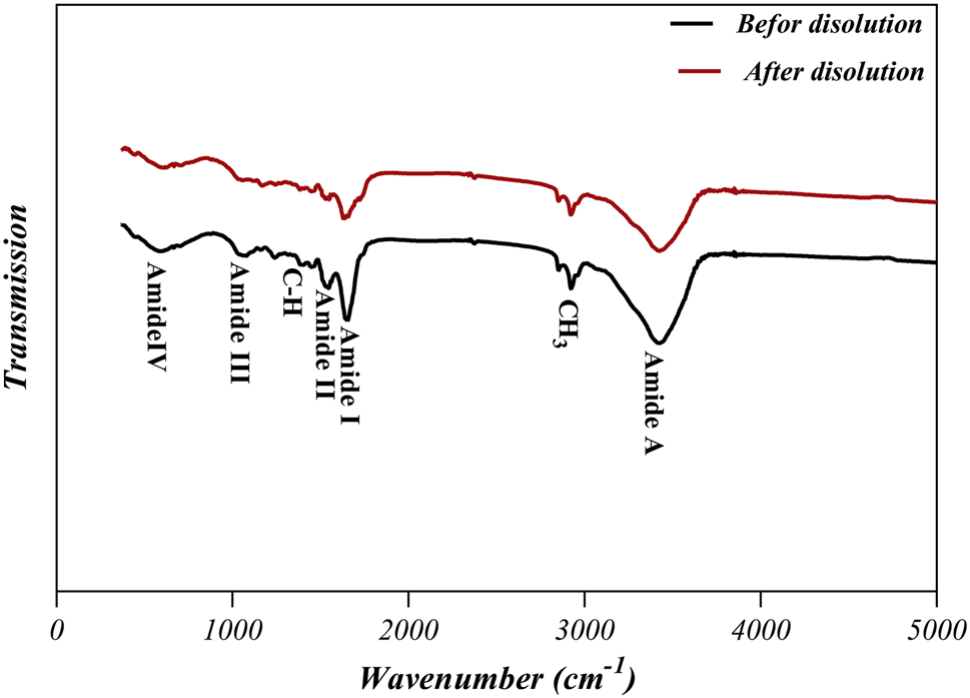
FTIR spectra of the amniotic membrane powder and the film resulting from its dissolution in the solvent FA/AA (70/30).

Figure 2 illustrates the FTIR spectra of amniotic membrane powder and film resulting from its dissolution in the solvent system FA/AA (70/30) for 24 h (labeled as HAMF). The absorption band around 1600–1700 cm^−1^ corresponds to the amide-I protein absorption band and the other absorption band around 1510–1580 cm^−1^ corresponds to the amide-II protein absorption band^18^. The peaks at around 1210–1300 and 1070–1080 cm^−1^ are attributed to amide III protein and also to the phosphodiester group of nucleic acids, glyco- and phospho-lipids^19^. The peak at 2960 cm^−1^ can be assigned to an asymmetry stretching mode of the CH_3_ group^20^. The band close to 1450 cm^−1^ is probably associated with the C-H bending modes and the amide-A band which is observed at 3300–3310 cm^−1^ is almost symmetric, suggesting that the amount of water must be low^21^. The peaks at 1398 and 640–650 cm^−1^ are attributed to the carboxylate ion and the C=O planar deformation vibration of amide IV, respectively^21,22^. Protein spectra are characterized by amide stretching and bending vibrations. As can be seen, there is no noticeable shift in the amide peaks among the spectrum patterns of the amniotic membrane powder and the HAMF film, indicating that there were no major changes in the functional groups and the nativity of the proteins such as collagen molecules before and after dissolution^21,23^.

### Characterization of the electrospinning solutions

**Figure 3:**
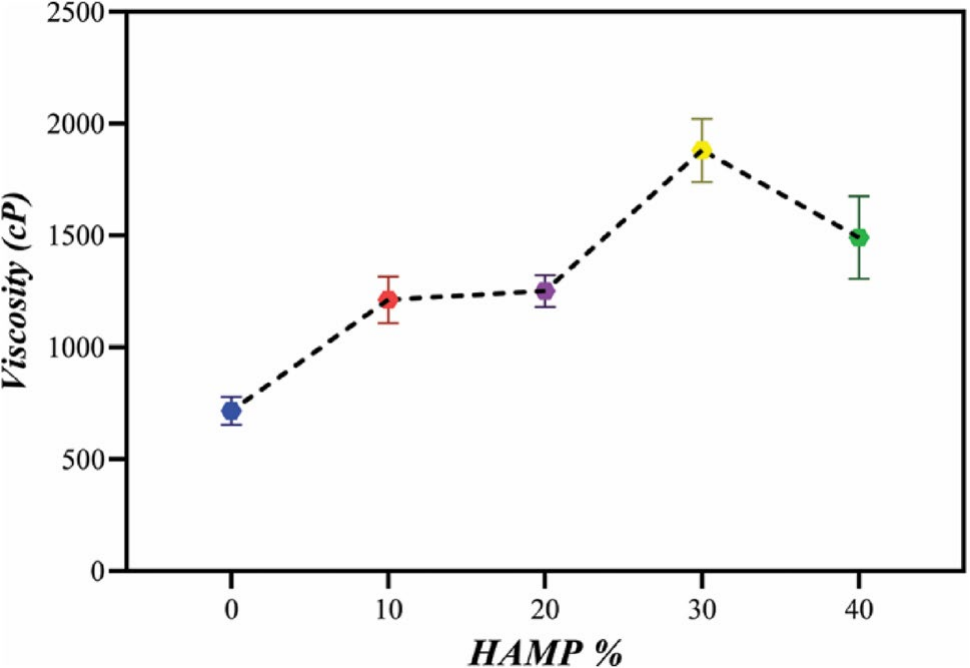
The viscosity of electrospinning solutions with different amounts of HAMP.

**Figure 4:**
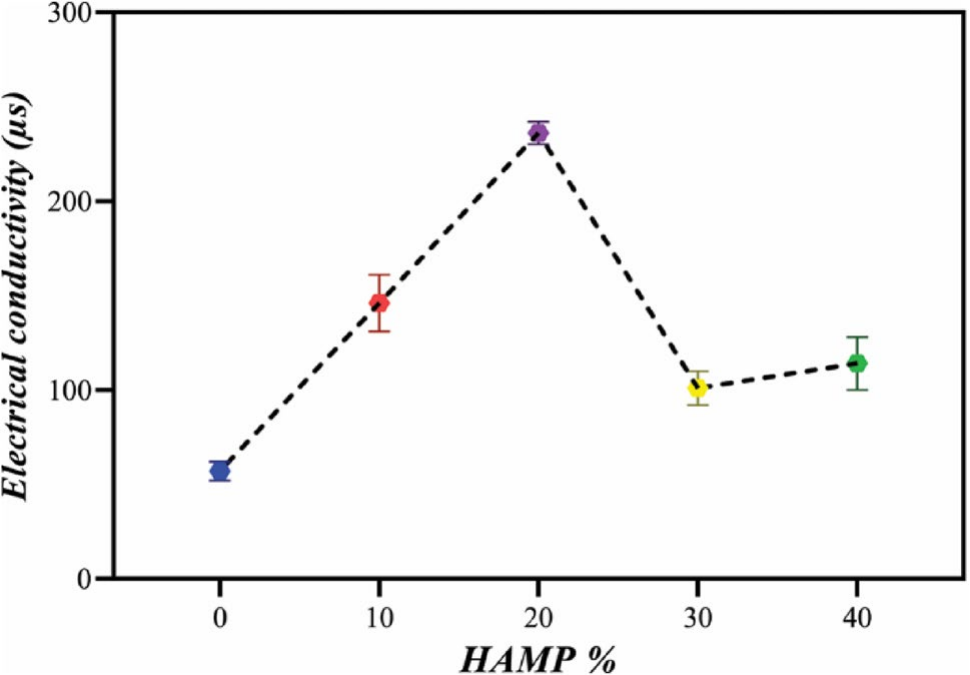
Electrical conductivity of electrospinning solutions with different amounts of HAMP.

The changes in viscosity and electrical conductivity of polymer solutions are shown in Figs. 3 and 4, respectively. Viscosity and electrical conductivity are the key factors that influence the formation of beaded and ribbon-like fibers during the electrospinning process^24,25^. As can be seen in Fig. 3, the viscosity increased with increasing the HAMP percentage in PCL-10HAMP and PCL-20HAMP solutions. The increase in viscosity is due to the simultaneous increase in the concentration of polymer solutions with the increase in the percentage of HAMP^26,27^. In the PCL-30HAMP solution, there is a sharp increase in viscosity. This phenomenon can be explained by the fact that at a weight percentage of 7.2 HAMP/PCL, the chains will tend to see each other more frequently and have a better chance at interacting which can result in chain aggregation. The effect of aggregation and increased interaction causes the viscosity of the suspension to rise^28,29^. The viscosity decreased in PCL-40HAMP compared to PCL-30HAMP. The viscosity of aggregated suspensions decreases much more rapidly due to the contribution of two parallel processes: (i) rearrangement of the particles inside the aggregates making aggregates more compact and (ii) formation of shear-induced structures at a macro-level^28^. As can be seen in Fig. 4, electrical conductivity increased with increasing the HAMP percentage in the PCL-10HAMP and PCL-20HAMP solutions. HAMP contains different types of collagens, laminin, and fibronectin. Collagen is a type of polyelectrolyte that can increase charge density, electrical conductivity^30,31^. In the PCL-30HAMP solution, there is a decrease in electrical conductivity due to the reduction of the number of load carriers caused by aggregation^32^.

### Characterization of the scaffolds

**Figure 5:**
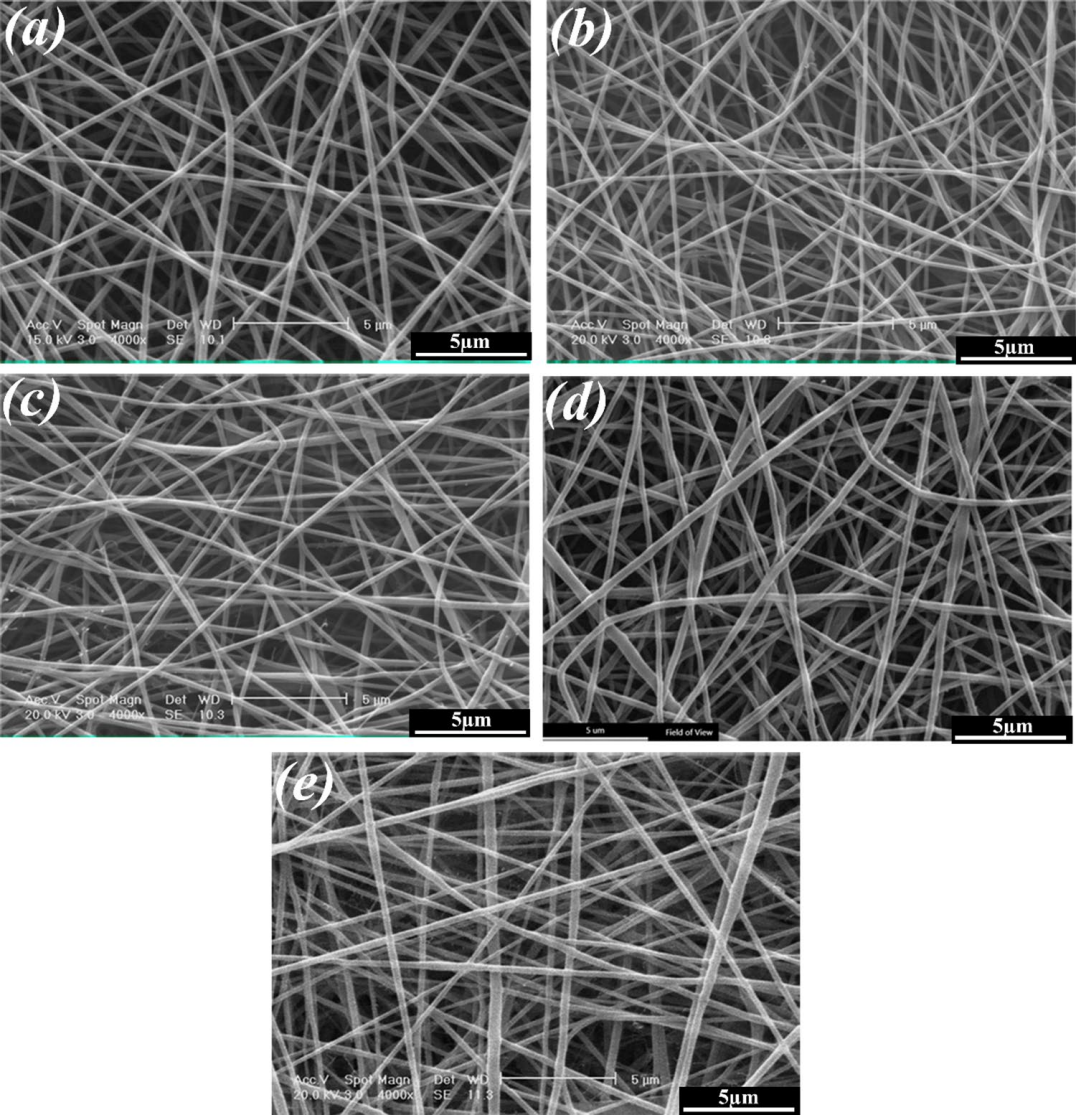
SEM images of the electrospun scaffolds: (**a**) PCL, (**b**) PCL-10HAMP, (**c**) PCL-20HAMP, (**d**) PCL-30HAMP, and (**e**) PCL-40HAMP.

**Figure 6:**
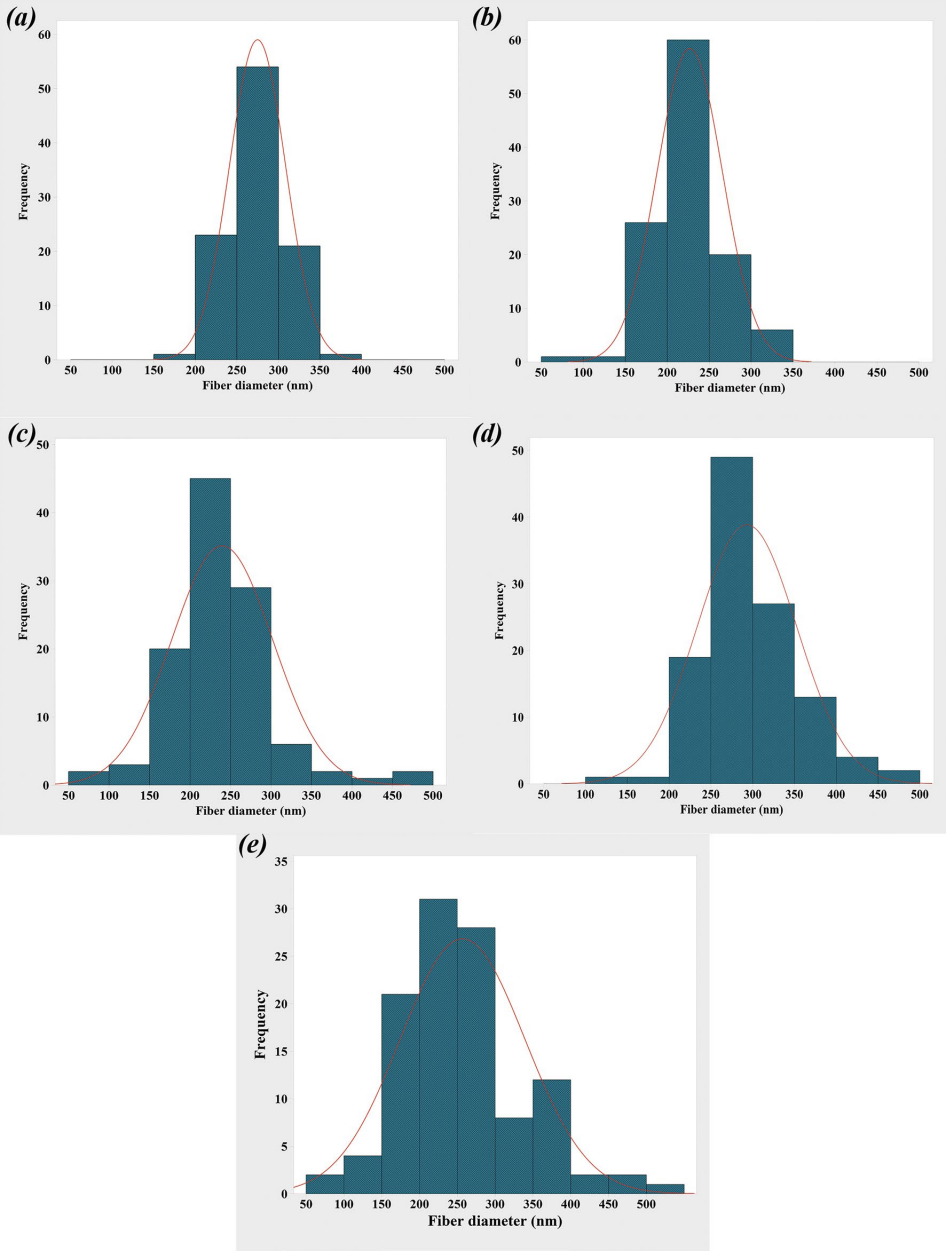
The histogram of the fiber diameter distribution and the curve of normal-log distribution of the electrospun scaffolds: (**a**) PCL, (**b**) PCL-10HAMP, (**c**) PCL-20HAMP, (**d**) PCL-30HAMP, and (**e**) PCL-40HAMP.

**Figure 7:**
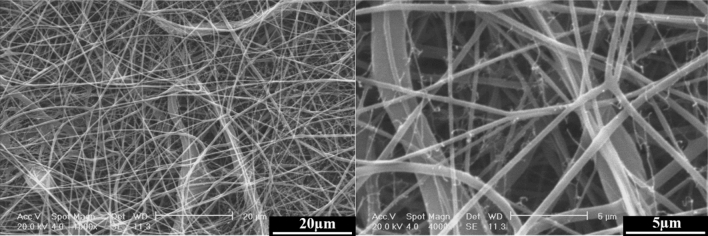
SEM images of the PCL-50HAMP scaffold, at different magnifications.

Figures 5 and 6 show the SEM images of the electrospun PCL, PCL-10HAMP, PCL-20HAMP, PCL-30HAMP, PCL-40HAMP scaffolds, and the histogram of the fiber diameter distribution. Figure 7 shows the SEM images of the PCL-50HAMP scaffold. As can be seen, fibers with a good morphology without any beads and defects were obtained in the PCL, PCL-10HAMP, PCL-20HAMP, PCL-30HAMP, and PCL-40HAMP scaffolds. But in the PCL-50HAMP sample, the fibers do not have a good morphology and a large amount of beads, drops, and flat fibers are obtained. This is probably due to the excessive increase in viscosity due to the increase in concentration^33,34^. In this study, as shown in Fig. 6 and Table 2, with the addition of HAMP in the PCL-10HAMP sample, the mean diameter of fibers decreased. This decrease is due to the high charge density on the surface of the solution, as described in Sect. 3.2, which can increase the voltage between the positive and negative poles, which forces the jet to spray lower diameter fibers. Several studies have reported that the combination of PCL and collagen reduces the fiber diameter^23,31^. With increasing the HAMP percentage in PCL-20HAMP and PCL-30HAMP, the viscosity and the average diameter of the fibers increased. The sharp increase in the mean fiber diameter in the PCL-30HAMP sample is due to a simultaneous decrease in the electrical conductivity in addition to an increase in viscosity (Figs. 3 and 4). In PCL-40HAMP, increasing the viscosity increased the average diameter of the fibers.

**Table 2:** The mean fiber diameter of the scaffolds.

	Scaffolds				
	PCL	PCL-10HAMP	PCL-20HAMP	PCL-30HAMP	PCL-40HAMP
Solution concentration (wt/v)	16.0	16.5	16.8	17.2	17.4
HAMP/PCL (wt%)	0.0	2.8	5.2	7.2	8.9
Mean fiber diameter (nm)	274.9 ± 33.8	226.9 ± 38.9	239.6 ± 62.5	293.2 ± 59.6	256.9 ± 82.5

The membranes containing HAMP displayed significantly increased fiber diameter range from 50 to 500 nm similar to fibers of Bruch’s membrane^35^. This increase in fiber diameter range is probably due to an increase in concentration. The increase in the solution concentration results in an increase in the viscosity and hence the increase of the viscoelastic forces which prevent the jet from being drawn by the Coulomb force resulting in a higher diameter of the fiber. On the other hand, increasing the concentration leads to an increase in load quantity and coulomb’s force^36^. The result of these two different factors produces a mixture of fibers with a diameter distribution.

**Figure 8:**
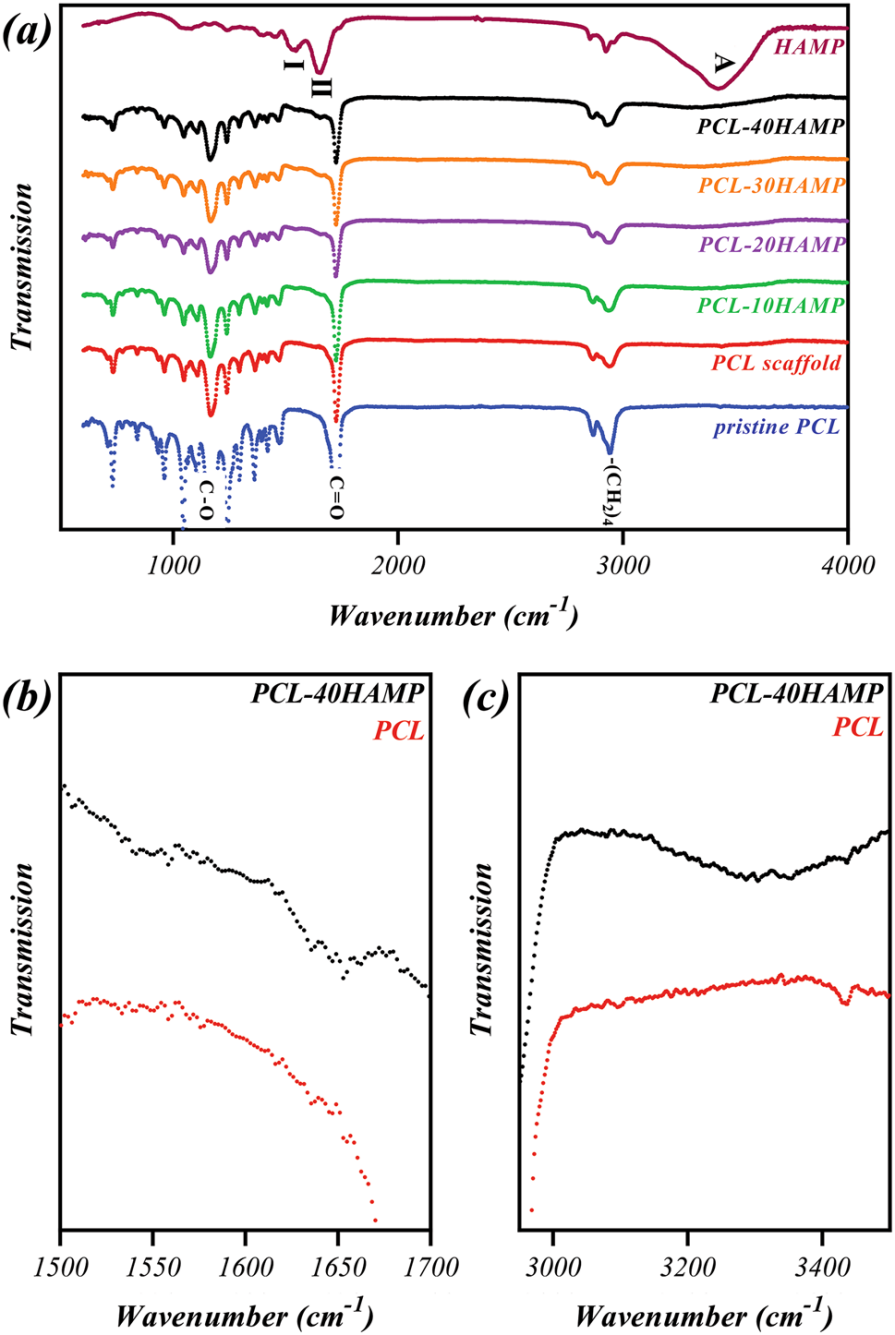
FTIR spectra of **(a)** PCL and PCL/HAMP scaffolds in the 600–4000 cm^−1^ region, **(b)** PCL and PCL-40HAMP scaffolds in the 1500–1700 cm^−1^ region, **(c)** PCL and PCL-40HAMP scaffolds in the 3000–3500 cm^−1^ region.

Figure 8 illustrates the FTIR spectra of the PCL and PCL/HAMP scaffolds. The presence of PCL was confirmed in all five types of the scaffolds based on all the characteristic groups associated with the PCL material: (i) the –(CH_2_)_4_ skeletal group in the 2850–3000 cm^−1^ region, (ii) the C=O bond around 1750 cm^−1^, and (iii) the C–O group in the 1150–1250 cm^−1^ region^37^. As it was detected, the spectra of PCL/HAMP with different proportions were closely similar, due to the small amount of HAMP added (Max weight percent 7.2 of HAMP/PCL). However, the amide-I, amide-II, and amide-A proteins absorption bands are observed at around 1510–1560, 1600–1700, and 3300–3310 cm^−1^, respectively. Also, the intensity of these peaks increased with increasing the HAMP content. These results revealed successful incorporation of the proteins within the PCL matrix evident from the amide-A, amide-I, and amide-II peaks. Zeybek et al.^38^. have also achieved similar results in electrospinning of the PCL/collagen scaffolds.

**Figure 9:**
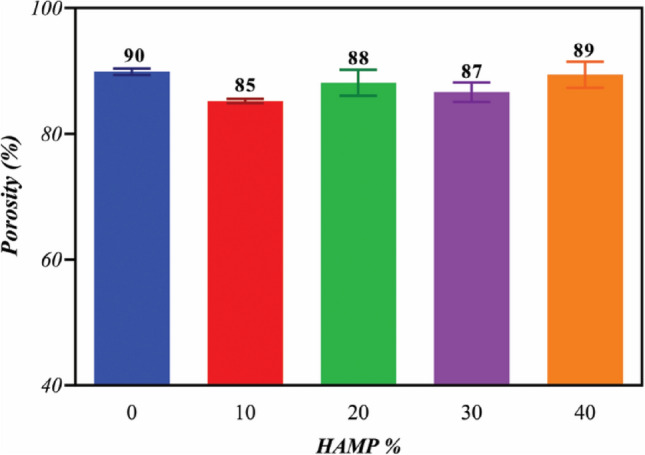
The porosity of the electrospun membranes with different amounts of HAMP.

The results of the porosity evaluation of the scaffolds are reported in Fig. 9. As can be seen, with increasing the percentage of HAMP, the percentage of porosity has not changed significantly, and the total porosities of all samples were between 85 and 90%. Porosity is a critical parameter for the selection of fibrous scaffolds for cell culture or tissue engineering and, usually, 60 to 90% porosity is considered appropriate for this purpose. The porosity of the scaffolds would appropriately support metabolite transport and provide a huge surface area for the RPE cell attachment^39–41^.

### Hydrophilicity

**Figure 10:**
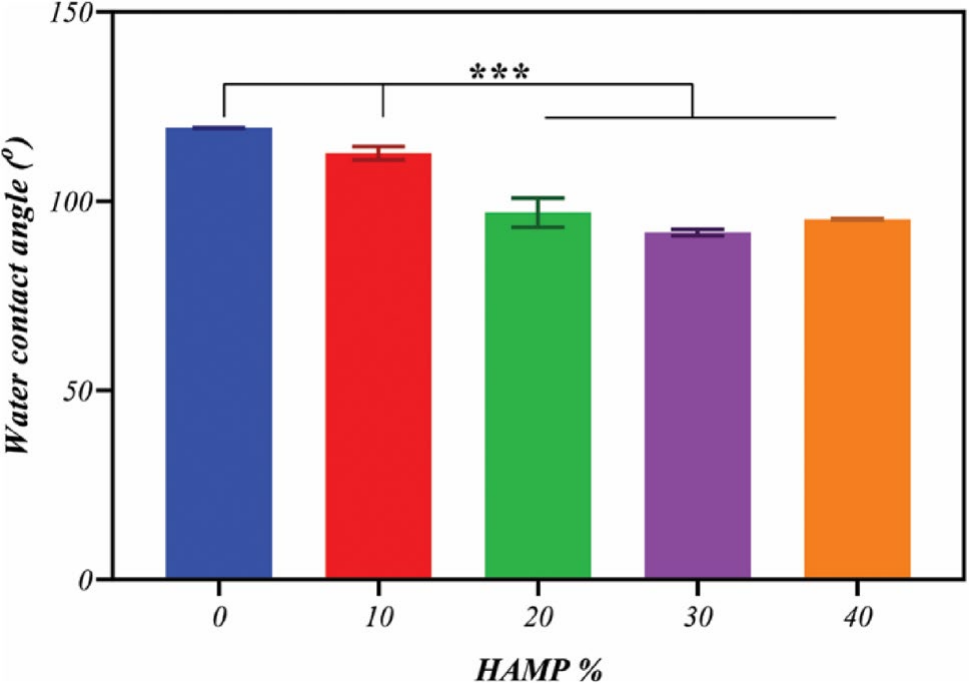
The water contact angle of fibrous scaffolds with different amounts of HAMP.

Figure 10 presents the water contact angle measurements for the PCL and PCL/HAMP scaffolds. The PCL electrospun scaffold was shown to have water contact angle values of approximately 119°, consistent with the hydrophobic nature of the polymer^42^. But it was revealed that by increasing the HAMP content of the scaffolds the water contact angle decreased, indicating higher hydrophilicity. HAMP is a combination of natural polymers such as different types of collagen, laminin, and fibronectin, and natural polymers are intrinsically hydrophilic because they are composed of polar molecules, such as polysaccharides^43^. The most hydrophilic scaffold was PCL-30HAMP having a contact angle of approximately 92°. However, the PCL-40HAMP scaffold had an insignificantly increased water contact angle (P < 0.05), approximately 95°. Previously, some studies have shown the effect of increased contact angles, most often for water, for the decreased fiber diameter^44,45^. This result was expected, as it is known that decreasing the surface fill fraction of a surface, or reducing the amount of polymer exposed at a given surface (and increasing the air fraction), will result in a higher apparent contact angle.

A contact angle below 90° means a hydrophilic surface and a contact angle above 90° corresponds to hydrophobic surfaces^46^. It can be considered PCL-30HAMP to be approximately hydrophilic.

### Mechanical properties

The Young’s modulus and max strain at failure of the electrospun scaffolds are summarized in Table 3. The addition of HAMP increased Young’s modulus in the PCL/HAMP scaffolds. This is probably due to the formation of newer hydrogen bonds between the amide groups of collagen and the carboxyl groups of PCL^37^. Another important feature that might influence the mechanical properties is the average diameters. Some reports have considered that thinner fibers have a higher elasticity modulus than thicker fibers^47,48^. This has been demonstrated by Aguirre-Chagala et al.^47^ for electrospun polycaprolactone/collagen/elastin fibers. This behavior matches our determined values, whereas the PCL-10HAMP scaffold had the highest Young’s modulus and the PCL-30HAMP scaffold had the lowest Young’s modulus. A physical interpretation of size-dependent nanofiber behavior is still a matter of debate and it has commonly been attributed to the surface tension effect. An increase in the E of the nano-objects can be described as a sum of the bulk and surface factors. The total energy U of deformed nanofibers includes the surface energy, which is responsible for the increase of E in the range of the small diameters^49^.

**Table 3:** The Young’s modulus and elongation-at-break values of the scaffolds.

	Scaffolds				
	PCL	PCL-10HAMP	PCL-20HAMP	PCL-30HAMP	PCL-40HAMP
Young’s modulus (MPa)	17.3 ± 0.3	28.6 ± 3.8	37.6 ± 3.1	25.6 ± 1.6	40.0 ± 5.3
Max strain at failure (%)	58 ± 3	113 ± 17	108 ± 5	38 ± 7	39 ± 3

These data indicate that the PCL-30HAMP scaffold is better in comparison to the other PCL/HAMP scaffolds, which decreases the likelihood of tissue injury and is mechanically robust enough to withstand manipulation during implantation, although no studies have been done to determine which mechanical properties correlate with successful cell growth, viability, functionality, and scaffold implantation^35^.

### Biodegradability analysis

**Figure 11:**
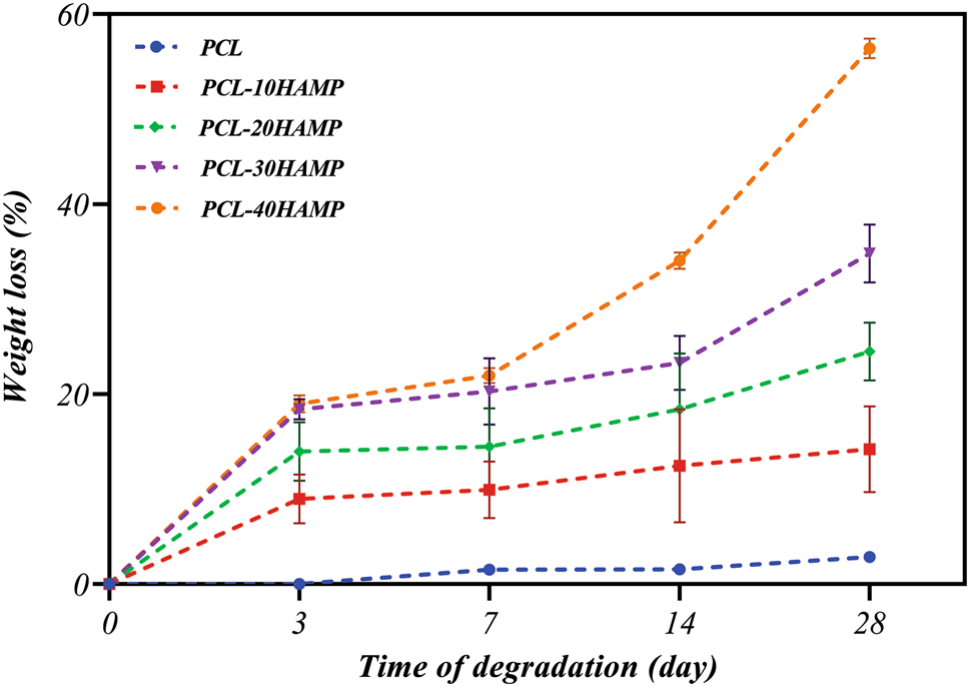
Weight loss percentage curves of the scaffolds after 28 days of immersion in the PBS solution.

Figure 11 shows the results of the biodegradability assessment of the scaffolds. The in vitro degradation of the scaffolds was estimated by weight loss measurements after 3, 7, 14, and 28 days of immersion in PBS at 37 °C, calculated by Eq. (1), and the profiles of the weight loss percentage are shown for all samples. No weight loss was observed in the PCL scaffolds until the third day, which is probably due to the initial phase of PCL biodegradability. Over time, its biodegradability increased very little, so that after 28 days, its weight loss percentage reached only 2.5%. But the rate of weight loss in all PCL/HAMP scaffolds during the first 3 days of biodegradation is very fast, being related to surface leaching, followed by a slower rate stage from deeper scaffolds layers. It is naturally anticipated that the very fast polymer leaching is due to surface erosion, and higher hydrophilicity, followed by much slower bulk erosion from the inner part of scaffolds^50^. In a typical process of bulk degradation, the weight could be first maintained followed by a sharp loss. During bulk degradation, water permeates into the bulk and induces hydrolysis and random chain fracture. Then the segmented chains diffuse out of the bulk leading to weight loss. It is easy to detect a sharp weight loss for the PCL/HAMP scaffolds, but not for PCL due to its slow degradation rate^51^. Also, with increasing the HAMP content in the scaffolds, degradability has increased, so that after 28 days the weight loss percentage of the PCL-10HAMP, PCL-20HAMP, PCL-30HAMP, and PCL-40HAMP scaffolds reached 14%, 24%, 35%, and 56%, respectively. In comparison with the PCL homopolymer which has a total degradation of 2 to 4 years^40^, the degradation rate of the PCL/HAMP scaffolds was considerably faster. The addition of HAMP to PCL could significantly affect the degradation rate of the scaffolds. This may be due to the presence of natural polymers in this powder as these polymers are degraded in biological systems by oxidation and hydrolysis^52^. The degradation rate depends on a variety of parameters such as crystallinity and molecular weight as well as the composition and morphological structure of the material which is used in the fabrication of the scaffolds^40^. It was pointed out that the main degradation mechanism of aliphatic polyesters in aqueous media is the cleavage of ester linkages via hydrolysis^53^. Degradation of poly(ε-caprolactone) is a bulk process that can be divided into two phases: (I) Molecular weight loss up to 5000 due to chain scission. No weight loss was observed during the initial phase of the biodegradation process, which covers a molecular weight range of 5000 to 200,000. (II) The phase is characterized by a decrease in the rate of chain scission and the onset of weight loss. The decrease in the rate of chain scission is associated with an increase in crystallinity since cleavage takes place in the amorphous region of the polymer. Weight loss has been attributed to an increased chain scission of low molecular weight (less than 3 000), polymer breakup to produce smaller particles^53^. Since the biodegradable property could be beneficial for tissue integration and avoidance of subsequent surgical removal of the scaffold, PCL/HAMP scaffolds are capable to support the growing cells during the monolayer formation and could be suitable substrates for regenerating the RPE layer.

### Characterization of ARPE cells

**Figure 12:**
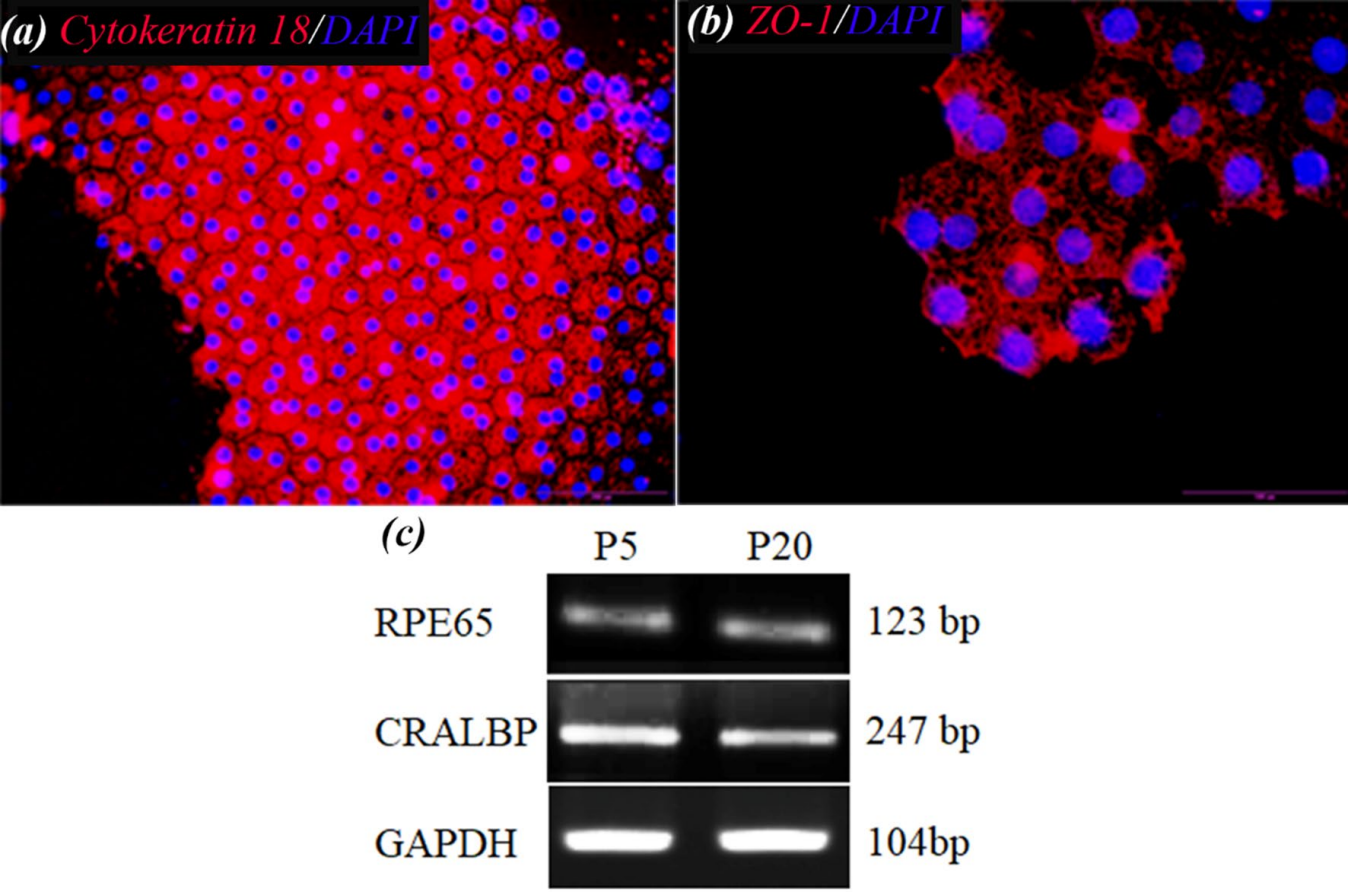
Immunostaining images of (**a**) Cytokeratin 18 and (**b**) ZO-1 of ARPE cells at day 7. These images indicated the monolayer sheet formation by ARPE cells but most of ZO-1 protein was observed in intracellular pools. (**c**) Expression of RPE65, and CRALBP genes in ARPE cells cultured for 7 days and from passage 5 (P5) and passage 20 (P20), detected by RT-PCR ((**c**) has been cropped and its original images are shown in the Supplementary Information file).

The results of immunocytofluorescence staining and RT-PCR are shown in Fig. 12. As it can be seen in Fig. 12a,b, the ARPE cells presented hexagonal morphology and expressed ZO-1 and cytoceratin18 as apical junctional complex and epithelial markers respectively. Most ZO-1 was found in intracellular pools. Rather than a continuous circumferential band, ZO-1 at the cell borders appeared as discontinuous puncta (Fig. 12b). Most importantly, RT-PCR analysis showed the expression of the visual cycle genes, RPE65 and CRLAPE that are expressed in RPE cells (Fig. 12c and Supplementary Fig. S1). CK18 is an intermediate filament protein and the major cytoplasmic component of epithelial cells. CK18 is primarily expressed in single-layered epithelial tissues or simple epithelium^54^. On the other side, ZO-1 is a membrane-associated tight junction adaptor protein that anchors the junctional macromolecular complexes to cytoplasmic actin^55^. ZO1 is important, but insufficient, criteria. ZO-1 is found in all cells where it participates in different types of cell junctions. ZO-1 is found in an apical junctional complex that lacks tight junctions^56,57^ Therefore, to accurately confirm the presence of a tight junction between the cells, we need other markers, including occludins and claudins or methods such as TEM or the transepithelial electrical resistance (TER).

Dunn et al.^58^ have demonstrated that ARPE-19 has structural and functional properties characteristic of RPE cells in vivo and suggest that this cell line will be valuable for in vitro studies of retinal pigment epithelium physiology. However, some reports demonstrated that with time in culture, the microheterogeneity of this cell line may change with continued passage in a way that depends on culture conditions. Most ZO-1 was found in intracellular pools. Rather than a continuous circumferential band, ZO-1 at the cell borders appeared as discontinuous puncta. This would add to the difficulty of comparing data among laboratories and be limitations of the working with this cell^56,59^.

### Study of cytocompatibility of the scaffolds

**Figure 13:**
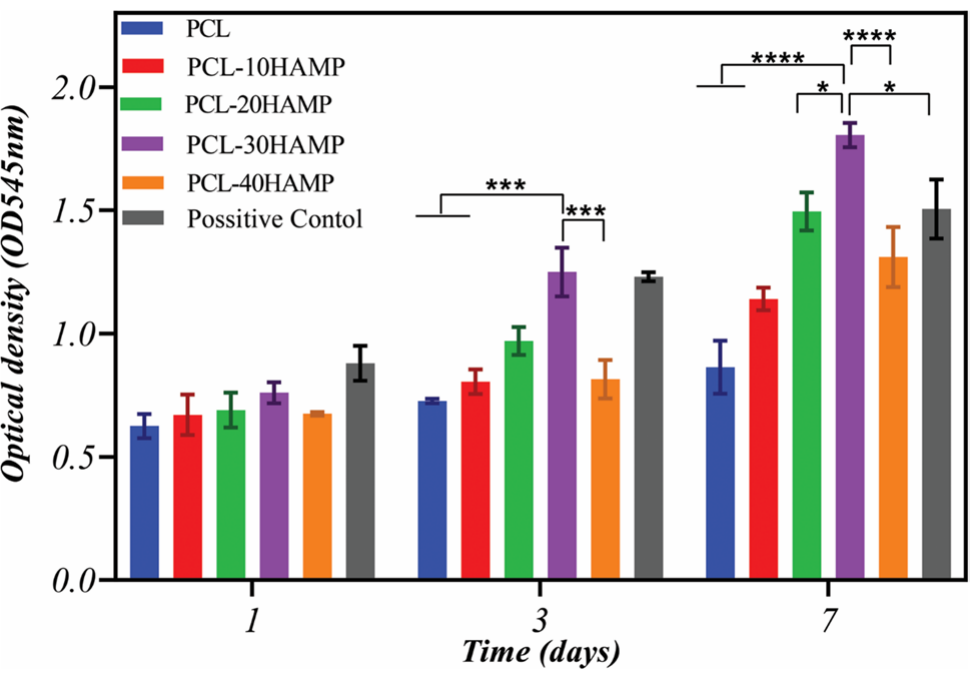
MTT results of the ARPE cells cultured on the tissue culture plate (as control) and electrospun scaffolds after 1, 3, and 7 days of cell seeding.

The comparison chart of the MTT assay performed on a control sample (TCP) and electrospun scaffolds is shown in Fig. 13. The absorption diagram over time shows that cell viability on all the scaffolds increased during cell culture. Then, HAMP has no toxic effect on ARPE cells. As can be seen, the amounts of adsorption on the first day in all the scaffolds and the control sample were not significantly different (p˂0.05), which indicates almost the same adhesion of cells on all samples. However, after seven days of cell culture, the adsorption rate in the PCL-30HAMP scaffold significantly increased compared with the other samples (p˂0.05), which indicates more growth and proliferation of the cells compared to other samples. Thus, the surface of these scaffolds is more suitable for cell growth and proliferation, which is consistent with the increase in hydrophilicity^60–63^. Also, superior cell attachment and spreading of the PCL-30HAMP compared with PCL-40HAMP are probably attributed to the higher surface roughness (p˂0.0001) due to larger fiber size^64^.

**Figure 14:**
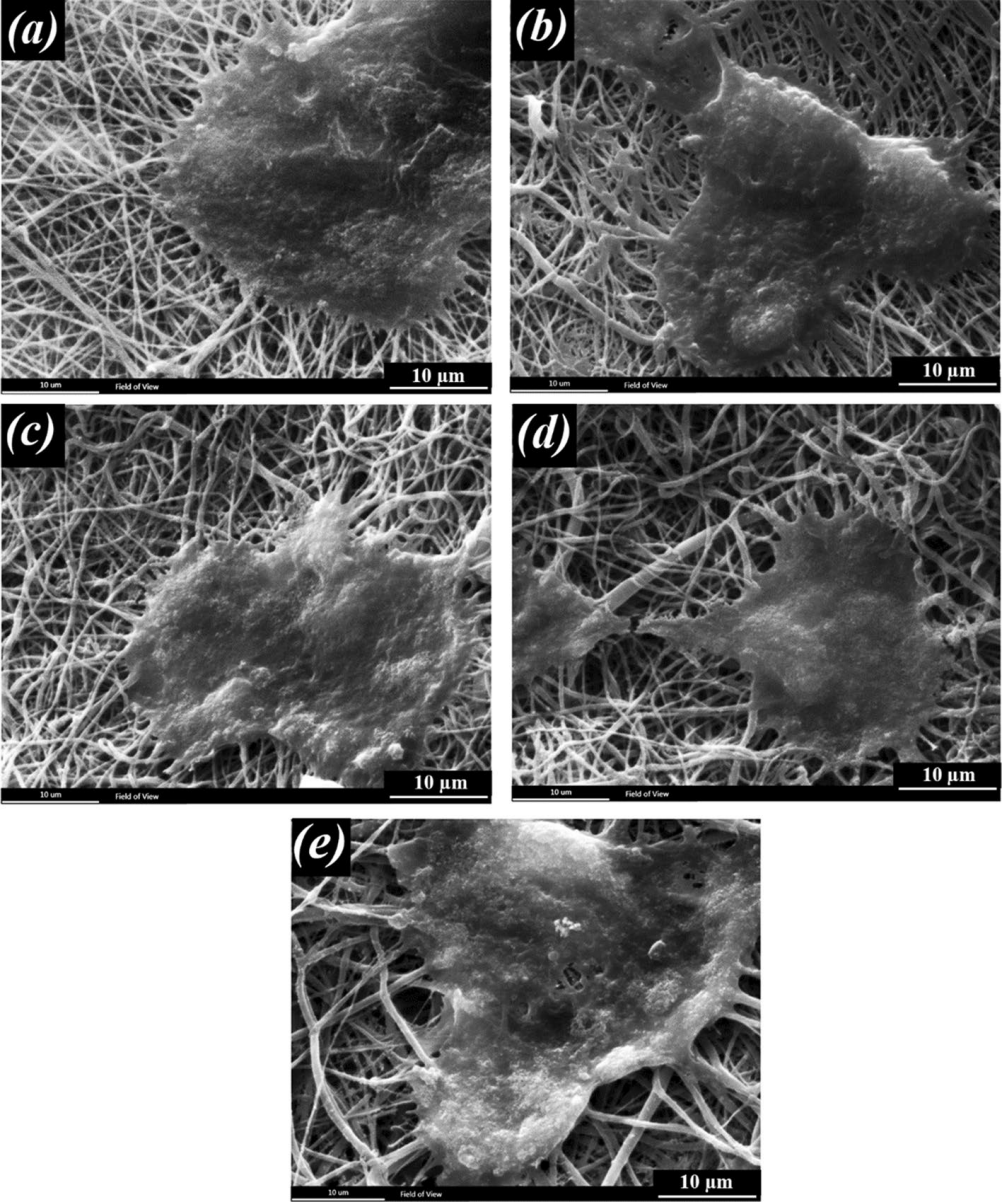
Morphology of the ARPE cells on the scaffolds after 1 day of culture, (**a**) PCL (**b**) PCL-10HAMP (**c**) PCL-20HAMP, (**d**) PCL-30HAMP, and (**e**) PCL-40HAMP.

**Figure 15:**
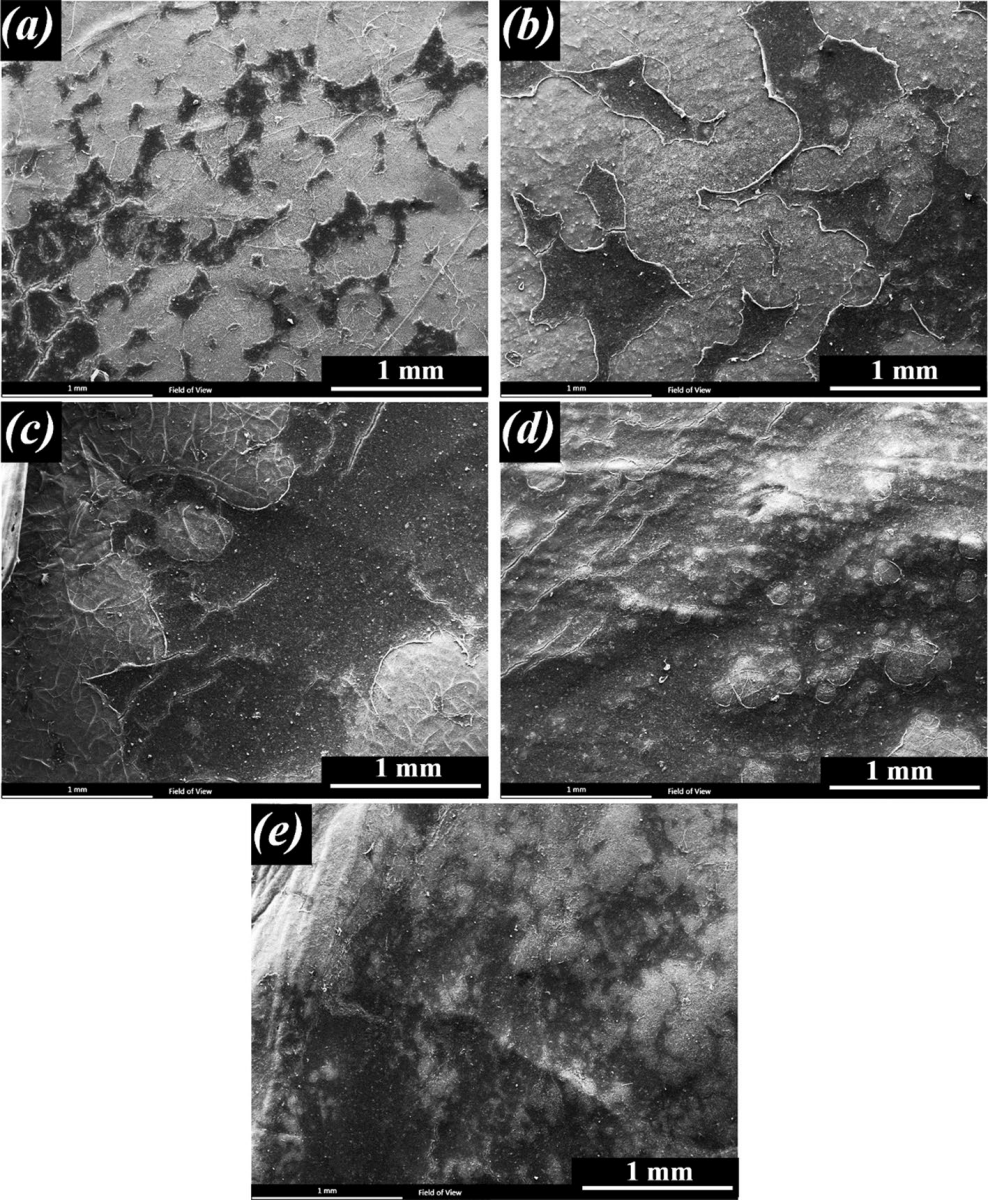
Morphology of the ARPE cells on the scaffolds after 7 day of culture (scale bar, 1 mm), (**a**) PCL (**b**) PCL-10HAMP (**c**) PCL-20HAMP, (**d**) PCL-30HAMP and (**e**) PCL-40HAMP.

Figures 14 and 15 show the interaction of ARPE cells with the prepared scaffolds 1 and 7 days following cell seeding, respectively. Figure 14 shows the excellent adhesion of the cells on all the scaffolds after 1 day and Fig. 15 shows the formation of a cell layer on the scaffolds after 7 days of cell culture.

**Figure 16:**
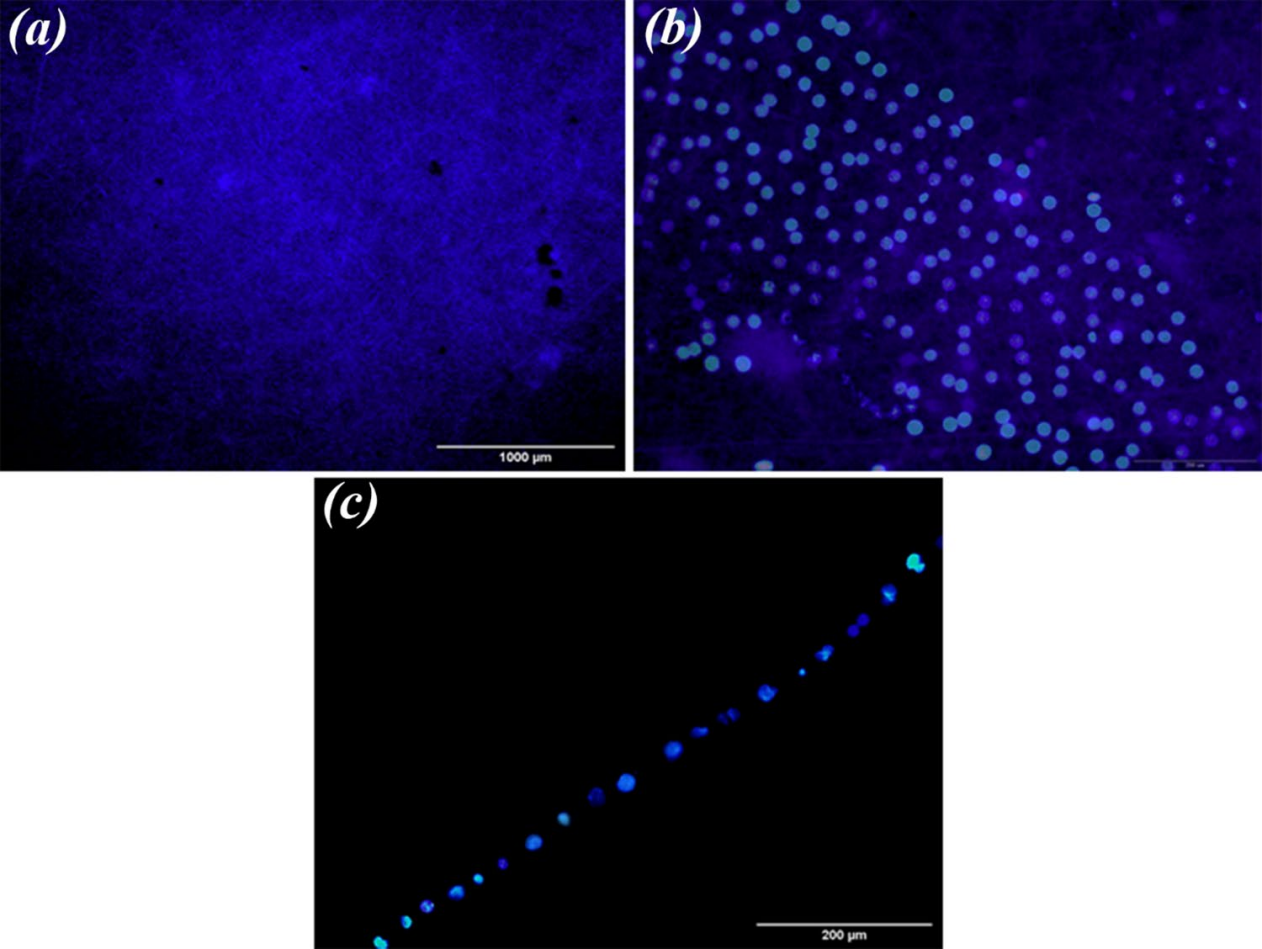
(**a**,**b**) Immunofluorescence images of nuclei (DAPI) in ARPE cells seeded on PCL-30HAMP scaffolds for 7 days. (**c**) Cross-sectional visualization of a monolayer of ARPE cells on PCL-30HAMP scaffolds with nuclei staining (DAPI) after 7 days.

Immunofluorescence images of nuclei (DAPI) in ARPE cells seeded on PCL-30HAMP scaffolds for 7 days are shown in Fig. 16. These images confirmed that the monolayer of the cells, due to no nuclei overlapping in the pictures.

RPE cell proliferation is influenced by cell density-dependent mechanisms, including cell–cell and cell-substrate contacts. Tight junction proteins such as claudins and occludins and gap junctional connexins are known to have a role in RPE cell–cell contact inhibition. The substrate also is another determinant of proliferation where integrins and other cell surface adhesion molecules mediate RPE cell-substrate adhesion and intracellular signaling^65^. As it can be seen in Fig. 15, over time, a high percentage of the surface is covered by cells due to cell growth and proliferation, indicating that the surface and environment of scaffolds are suitable for cell growth and proliferation. However, the rate of cell growth and proliferation is significant on the surface of all the scaffolds one week after culture. Complete cell coverage and cell sheet formation are visible in the PCL-30HAMP sample. This indicates that the surface and environment of this scaffold are more suitable than other scaffolds. These results may be due to its higher surface hydrophilicity and roughness than other scaffolds^66^ and are in good agreement with the results of the MTT evaluation.

The molecules of the HAM extracellular matrix, mainly fibronectin, laminin-1, laminin-5, collagen type-I, III, IV, V, and VII promote cell adhesion^67^. Most proteins (e.g., collagen, fibronectin or laminins) contain pro-adhesive sequences, such as RGD, PHSRN, YIGSR, or IKVAV. The combination of these proteins with polymer scaffolds results in the enrichment of biomaterial surfaces with beneficial binding sites. It is important which they are recognized by many kinds of cells (mainly via integrin receptors on the cell membrane). Consequently, such recognition promotes cell attachment and proliferation^68^. It is important to point out that according to the obtained results the initial adhesions of the cells are affected by the surface roughness and hydrophilicity of the material. The higher rate of cell adhesion and the shorter time of cell diffusion helps in increasing cell proliferation.

## Conclusion

PCL/HAMP fibrous scaffolds with high porosity, above 85%, were successfully prepared via electrospinning using the unconventional solvent system FA/AA and optimized based on the formation of intact cell sheets and full coverage of cells on the 7th day for AMD treatment. The results showed that with increasing the amount of HAMP, the diameter range of fibers increased (50 to 500 nm). Also, hydrophilicity and degradation rate significantly improved. Reasonable ARPE cells adhesion, viability, and morphology (monolayer) on the PCL-30HAMP scaffold suggested these scaffolds as a matrix that can support the proliferation of the RPE. The results showed that PCL-30HAMP scaffold with optimum porosity, degradation rate, and biocompatibility can be a good candidate for cell replacement therapies. However, further investigations are needed to confirm and explain these findings. Studies with ARPE 19 cells should use a differentiation medium and allow 4–6 weeks for junctional complexes to mature^56^. Further tests of this scaffold would use more authentic cultures of RPE that were derived from primary, or stem cell-derived, cultures of RPE^69^.

## Supplementary Information

Supplementary Information.

## Data Availability

All data generated and/or analyzed during the current study are available from the corresponding author on reasonable request.
