# Supplementary material for: Development of an electrospun poly(ε-caprolactone)/collagen-based human amniotic membrane powder scaffold for culturing retinal pigment epithelial cells
Source: Sci Rep. 2022 Apr 19;12:6469. doi: 10.1038/s41598-022-09957-5 (PMC9018818; doi:10.1038/s41598-022-09957-5)
Supplement: Supplementary file 1 — Supplementary Information. [file 41598_2022_9957_MOESM1_ESM.docx]

| 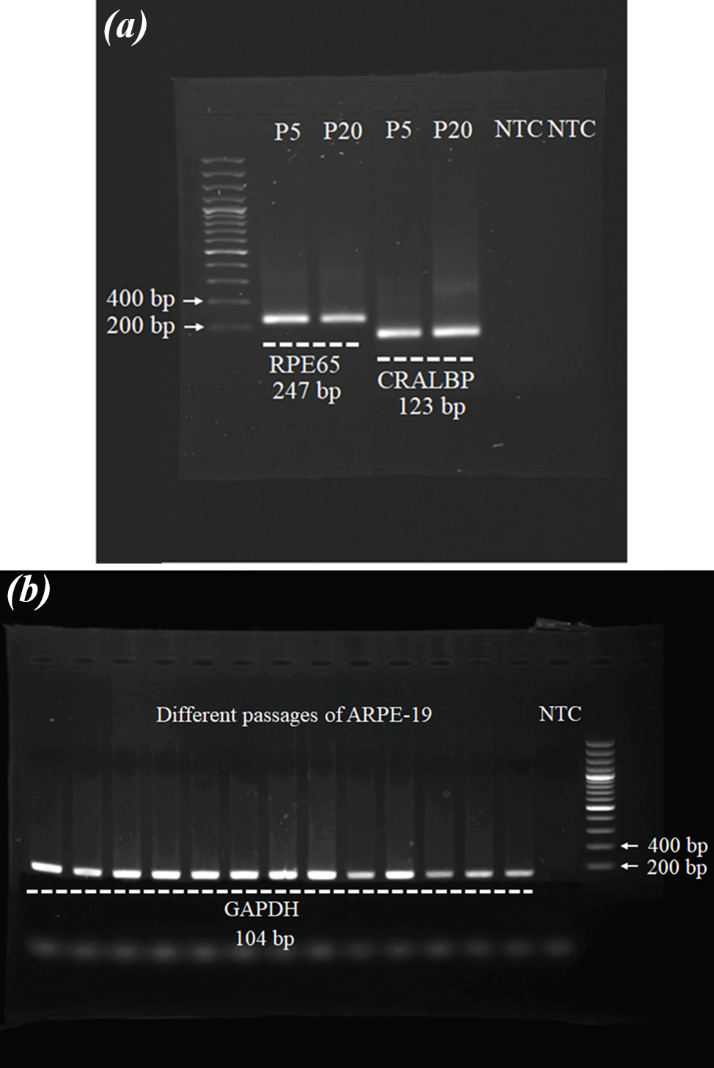 |
| --- |
| **Fig. S1.** Images of the expression of RPE genes in ARPE-19 cells. (a) RNA was isolated from the cells of passages 2 and 20 and analyzed by RT-PCR using specific primers for RPE65 and CRALBP mRNA. Non-template control (NTC) was considered as a negative control. (b) GAPDH was used as a housekeeping |
